# Elevated GLUT4 and glycogenin protein abundance correspond to increased glycogen content in the soleus muscle of *mdx* mice with no benefit associated with taurine supplementation

**DOI:** 10.14814/phy2.13596

**Published:** 2018-02-27

**Authors:** Robert G. Barker, Barnaby P. Frankish, Hongyang Xu, Robyn M. Murphy

**Affiliations:** ^1^ Department of Biochemistry and Genetics La Trobe Institute for Molecular Science La Trobe University Melbourne Victoria Australia

**Keywords:** Duchenne muscular dystrophy, *mdx* mouse, taurine, skeletal muscle, glycogen, metabolism

## Abstract

Duchenne muscular dystrophy (DMD) patients and the dystrophic *mdx* mouse have an elevated demand for ATP requiring processes, including Ca^2+^ regulation and skeletal muscle regeneration. As a key substrate for cellular ATP production, altered glycogen metabolism may contribute significantly to dystrophic pathology and explain reports of mild glucose intolerance. We compare the soleus and extensor digitorum longus (EDL) muscles of the *mdx* mouse during active muscle necrosis (at 28 days) and at 70 days where pathology is stable. We further investigate the impact of taurine (tau) on dystrophic glycogen metabolism to identify if the benefit seen with tau in a previous study (Barker et al. 2017) was in part owed to altered glycogen handling. The soleus muscle of 28‐ and 70‐day‐old *mdx* mice had elevated glucose transporter type 4 (GLUT4), glycogenin protein abundances and glycogen content compared to WT (C57BL10/ScSn) controls. *Mdx* tau mice exhibited modestly reduced glycogen compared to their respective *mdx* group. The EDL muscle of 28 days *mdx* tau mice had a ~70% increase in glycogenin protein abundance compared to the *mdx* but 50% less glycogen content. A twofold greater phosphorylated glycogen synthase (p‐GS) and glycogen phosphorylase (p‐GP) protein abundance was observed in the 70‐day‐old *mdx* soleus muscle than in the 28‐day‐old *mdx* soleus muscle. Glycogen debranching enzyme (GDE) protein abundance was elevated in both 28‐ and 70‐day‐old *mdx* soleus muscles compared to WT controls. We identified an increase in proteins associated with glucose uptake and utilization specific to the predominantly slow‐twitch soleus muscle of *mdx* mice regardless of age and that taurine affords no obvious benefit to glycogen metabolism in the *mdx* mouse.

## Introduction

Approximately 1:3600–6000 males are born with the fatal neuromuscular disorder Duchenne muscular dystrophy (DMD) (Bushby et al. 2010). DMD is characterized by the absence of the cytoskeletal protein dystrophin, which anchors the contractile apparatus of the skeletal muscle to the extracellular matrix providing myofibers with stability during contraction. Without dystrophin, myofibers become highly susceptible to contraction‐induced damage and/or ion channel dysfunction leading to Ca^2+^ influx and the subsequent activation of proteases and myofiber necrosis (for recent review, see Allen et al. (2016)).

Despite the increased demand for ATP requiring processes such as Ca^2+^ regulation and skeletal muscle regeneration, DMD patients and the similar dystrophin‐deficient *mdx* mouse model exhibit impaired energy metabolism with up to 50% reduction in resting ATP levels reported in dystrophic skeletal muscle (Austin et al. 1992; Cole et al. 2002). DMD patients have been reported to exhibit a decreased resting energy expenditure and this is likely due to the inability to perform functional exercise (Shimizu‐Fujiwara et al. 2012). Critically, the energy required for skeletal muscle regeneration, especially during the years when aggressive muscle deterioration is taking place in DMD, is unknown. Certainly, increased energy expenditure is evident during chronic onset of hind limb muscle necrosis seen in the *mdx* mouse at 3–4 weeks of age (Grounds et al. 2008; Radley‐Crabb et al. 2014; Barker et al. 2017). While this energy demand is decreased in older *mdx* mice (12–14 weeks of age) as the severity of the dystropathology reduces to low grade yet persistent muscle damage, energy requirements remain elevated compared to wild‐type controls (Grounds et al. 2008; Radley‐Crabb et al. 2014; Barker et al. 2017).

Little is known about glycogen metabolism in DMD (for review, see Cruz Guzmán et al. (2012)). As one of the major sources of metabolic fuel for skeletal muscle function, regeneration and growth (Melendez et al. 1997), altered glycogen metabolism may have a role in explaining the reduced energy metabolism seen with DMD. Glycogen is a densely branched polymer of glucose centered on the autoglucosylation of a core protein, glycogenin (Campbell and Cohen 1989). Glycogen is stored in the skeletal muscle and liver, and glycogen molecules are grown by the addition of glucose in times of energy and nutrient surplus by the actions of glucose transporter type 4 (GLUT4), glycogen synthase (GS, α‐1,4‐glycosidic linkages), and glycogen branching enzyme (GBE, α‐1,6‐glycosidic linkages), and utilized in times of caloric deficit or demand by glycogen phosphorylase (GP, α‐1,4‐glycosidic linkages) and glycogen debranching enzyme (GDE, α‐1,6‐glycosidic linkages) (Frosig and Richter 2009). Aberrant glycogen metabolism along with glucose intolerance has been reported in the *mdx* mouse previously, with alterations found in both the activity and abundance of glycogen associated proteins (Stapleton et al. 2014); however, the significance of age, which reflects the severity of the pathology as well as muscle specific differences remain undefined.

Interestingly in DMD patients, despite up to 75% reduction in skeletal muscle mass by their early teens (Letellier et al. 2013), patients are reported to have only mild glucose intolerance (Freidenberg and Olefsky 1985). Considering that the skeletal muscle is one of the key glucose stores within the body, these findings suggests a functional adaptation in the skeletal muscle or liver to enable adequate glucose clearing from the bloodstream in DMD patients. It may also be possible that circulating glucose is used for energy directly, although this is unlikely considering reports of decreased resting energy expenditure in the *mdx* model (Shimizu‐Fujiwara et al. 2012).

Found ubiquitously in all mammalian cells, the amino acid taurine has extensively been characterized as vital for healthy muscle function and development (Huxtable 1992; De Luca et al. 2015). Dystrophin deficiency has been shown to impact taurine metabolism (Terrill et al. 2015), and in the *mdx* mouse, taurine levels have been found to be reduced both before and during the onset of the severe dystropathology in the skeletal muscle (21–28 days of age), returning to endogenous levels as the pathology stabilizes into adulthood (McIntosh et al. 1998; Griffin et al. 2001). Taurine has previously been shown to enhance glycogen repletion in the *tibialis anterior* muscle of mice postexercise (Takahashi et al. 2014, 2016), with results suggesting taurine reduced the use of carbohydrates as fuel allowing greater glycogen storage. This stored glycogen may be of benefit to regenerating skeletal muscle in *mdx* mice, particularly when aggressive muscle deterioration is taking place, although to date this has not been examined.

Previously, we identified prenatal taurine supplementation reduced the severity of myofiber necrosis and increased in situ strength in *mdx* mice at 28 days of age (Barker et al. 2017); however, we could not identify the pathway by which taurine was producing this benefit. Using the same mice used in our previous work (Barker et al. 2017), here, we investigate the hypothesis that taurine supplementation benefited the 28 days *mdx* mouse through an alteration in glycogen metabolism. We do this by comprehensively characterizing the abundance of glycogen‐associated proteins (glycogenin, GS, GBE, GP, and GDE) as well as GLUT4 in the fast‐ and slow‐twitch skeletal muscles of the *mdx* mouse. We further characterize glycogen metabolism in the 70 days *mdx* mouse in order to identify specific changes with age/or pathological severity.

## Materials & Methods

### Animals and supplementation

All procedures in this study were approved by the La Trobe University Animal Ethics Committee (AEC 12–31, 13–48). Muscle samples from the same mice used in our previous work (Barker et al. 2017) were utilized in the current study. A total of 35 *mdx* and 24 wild‐type (WT, C57/BL10ScSn) male mice were used for experiments. Experimental animals were bred at the La Trobe Animal Research and Teaching Facility using breeding pairs obtained from the Animal Resource Centre (Western Australia, Australia). The offspring of WT and *mdx* mice had free access to standard rodent chow and water ad libitum, and were utilized for experimentation at either 28 ± 1 or 70 ±1 days of age. Maximum litter size grown to maturity was six males. There was no significant difference between body weights of each age group at the age of experimentation (Barker et al. 2017). *Mdx* taurine (tau) breeders and subsequent offspring were supplemented with continuous access to taurine (2.5% wt/vol)‐enriched drinking water, with breeders beginning supplementation at least 2 weeks prior to mating. This dosage of supplementation has been demonstrated previously to elevate skeletal muscle taurine content in *mdx* mice (Barker et al. 2017).

### Muscle dissection

Mice were anesthetized with an intraperitoneal injection of 10 *μ*L·g^−1^ Nembutal (Sodium Pentobarbitone) and kept unresponsive to tactile stimuli while the soleus and extensor digitorum longus (EDL) muscles were excised. Mice were then killed by cardiac excision. Excised muscled were blotted clean on a filter paper (Whatman No.1) and weighed, before being snap frozen in liquid nitrogen and stored at −80°C until analysis.

### Western blotting

Frozen transverse EDL and soleus cryosections were cut from the midpoint (~30 × 10 *μ*m for 28 days animals and ~20 × 10 *μ*m for 70 days animals) and immediately placed into very low Ca^2+^ intracellular physiological buffer (PB) containing (mmol·L^–1^): 129 K^+^, 36 Na^+^, 1 free Mg^2+^ (10.3 total Mg^2+^, 90 HEPES, 50 EGTA, 8 ATP, 10 CP, pH 7.10, and an osmolality of 295 ±10 mosmol·kg^−1^ H_2_O^−1^) containing α‐amylase (A4268, Sigma‐Aldrich) to aid glycogen breakdown and liberation of tightly associated proteins (Xu et al. 2015). Samples were incubated for 30 min at 39°C, vortexed at 5 min intervals, and diluted 2:1 with 3× SDS solution (0.125 mol·L^–1^ Tris‐HCI, 10% glycerol, 4% SDS, 4 mol·L^–1^ urea, 10% mercaptoethanol, and 0.001% bromophenol blue, pH 6.8) and kept at room temperature for a further 30 min, vortexed at 5 min intervals and stored at −80°C until analyzed. Aliquots of each soleus and EDL samples were pooled together and used to create a calibration curve for that respective muscle that was run on every gel, allowing comparisons of whole muscle homogenates across gels (Mollica et al. 2009; Murphy and Lamb 2013). Total protein from each sample was initially separated on 4–15% gradient Criterion TGX Stain Free gels (BioRad, Hercules, CA), and following ultraviolet activation using a Stain Free Imager (BioRad), the densities of the total lanes was obtained (Image lab software v 5.2, BioRad) and used to determine equal loading for subsequent western blotting.

The western blotting protocol was similar to that described previously (Mollica et al. 2009; Murphy and Lamb 2013; Barker et al. 2017). Semiquantitative western blotting was performed to determine the protein abundance of GS, phosphorylated GS (p‐GS), GBE, GP, phosphorylated GP (p‐GP), GDE, and GLUT4 proteins. Briefly, a similar amount of protein from skeletal muscle samples was separated on 4–15% gradient Criterion TGX Stain Free gels (BioRad, Hercules, CA). Prior to transfer, gels were imaged with a Stain Free Imager (BioRad) for total protein which was quantified for each sample using Image lab software (v 5.2, BioRad). Following this, using a wet transfer protocol, protein was transferred onto a nitrocellulose membrane at 100 V for 30 min. Following the transfer, the gel was imaged again and the membrane incubated in Pierce Miser solution (Pierce, Rockford, IL) for ~10 min and then blocked in 5% skim milk powder in 1% Tris‐buffered saline‐Tween (TBST) for ~2 h at room temperature. Following blocking, membranes were incubated in primary antibody overnight at 4°C and 2 h at room temperature. Antibody details and dilutions: anti‐GS (rabbit monoclonal, 1:1000, Epitomics, 1720‐1); anti‐phos‐GS‐Ser641 (rabbit monoclonal, 1:2500, Epitomics 1919‐1 [detects phosphorylation at Ser641]), anti‐GBE (rabbit polyclonal, 1:5000, DS*), anti‐GP (rabbit polyclonal, 1:1000, David Stapleton [DS]), anti‐phos‐GP (rabbit polyclonal, 1:1000, DS), anti‐GDE (rabbit polyclonal, 1:1000, DS), anti‐glycogenin (rabbit polyclonal, 1:1000), and anti‐GLUT4 (rabbit polyclonal, 1:1000, ThermoScientific, PA1‐1065). Primary antibodies raised against GBE, GP, and GDE have been previously described (Parker et al. 2007; Murphy et al. 2012). Primary antibodies raised against glycogenin as described (Ryu et al. 2009). *Mdx* mice muscle samples were probed for dystrophin (mouse monoclonal, 1:500, Development Studies Hybridoma Bank [DSHB], MANDYS1 clone 3B7) to confirm the absence of this protein. All antibodies were diluted in 1% bovine serum albumin (BSA) in phosphate‐buffered saline with 0.025% Tween (PBST).

After washing, membranes were incubated with a secondary antibody (goat anti‐mouse IgG or IgM, goat anti‐rabbit IgG, HRP conjugated, 1:60,000) and rinsed in TBST. Bands were visualized using a West Femto chemiluminescent substrate (ThermoScientific, IL) and densitometry was performed using Image Lab software (BioRad). Images were taken using Image Lab software (BioRad). The positions of molecular mass markers were captured under white light, and then chemiluminescent imaging was taken without moving the membrane. Total protein and specific protein densities were each expressed relative to their respective calibration curves and subsequently each protein was normalized to the total protein content (Murphy and Lamb 2013). Data were then expressed relative to the average of the 28 days WT on a given gel. Representative blots for figures have been created by superimposing blots on top of the molecular mass maker, with black lines indicating noncongruent images from the same probe.

### Skeletal muscle glycogen content analysis

Glycogen content was analyzed similar to that described previously by Xu et al. (2015). The soleus and EDL muscles were homogenized in a very low [Ca^2+^] intracellular relaxing physiological solution containing (in mmol·L^–1^): 129 K^+^, 36 Na^+^, 1 free Mg^2+^ (10.3 total Mg^2+^), 90 HEPES, 50 EGTA, 8 ATP, 10 CP, pH 7.10, and an osmolality of 295 ± 10 mosmol·kg·H_2_O^−1^) to give a 50 *μ*g·*μ*L^−1^ wet weight tissue solution. Potassium hydroxide (100 *μ*L of 4 mol·L^‐1^ KOH) was added to 50 *μ*L muscle homogenate before samples were heated at 70°C for 30 min. After heating, the pH of the muscle preparation was brought to 4.5 with acetic acid. Four units of amyloglucosidase (A1602, Sigma‐Aldrich) was added to the solution before being incubated at 55°C for a further 2 h. During sample incubation, glucose oxidase/peroxidase (PGO enzyme) was prepared by mixing one capsule of PGO (P7119, Sigma‐Aldrich) with 1 mL dianisidine/dihydrochloride (D3252, Sigma‐Aldrich) made to a final volume of 50 mL volume with Milli‐Q H_2_O, and kept in the dark according to the manufacturer's instructions. PGO enzyme (240 mL) was then added to each sample as well as glucose standards with a calibration range of 7–500 *μ*g·mL^−1^ glucose and a blank (Milli‐Q H_2_O). After 5–10 min and within 30 min, the absorbance of samples and standards were read on a UV–VIS spectrophotometer (SpectraMax M5, Molecular Devices, Australia) at 450 nm. The glucose content of a given sample was determined against the glucose calibration curve.

### Muscle Histology

Transverse cryosections (10 *μ*m) were cut from the midpoint of the soleus and EDL muscles, and mounted on positively charged microscope slides (Lomb Scientific) for qualitative analysis. Both EDL and soleus slides used for direct comparison were periodic acid–Schiff stained (PAS) simultaneously, to ensure all samples were treated identically and stain intensity would be similar. Images were taken using a Motic BA310 microscope mounted with a Moticam 5 camera running Motic image plus 2.0 software (Motic, China).

### Statistics

All data are presented as mean ± standard deviation (SD) unless stated otherwise. Comparisons between multiple groups were performed using a one‐way analysis of variance (ANOVA), with Holm–Sidak's post hoc analyses. All statistical analysis was performed using GraphPad Prism v 6. Significance was set at *P *< 0.05.

## Results

### α‐Amylase cleaves glycogen synthase but has no effect on total protein abundance

**Figure 1:**
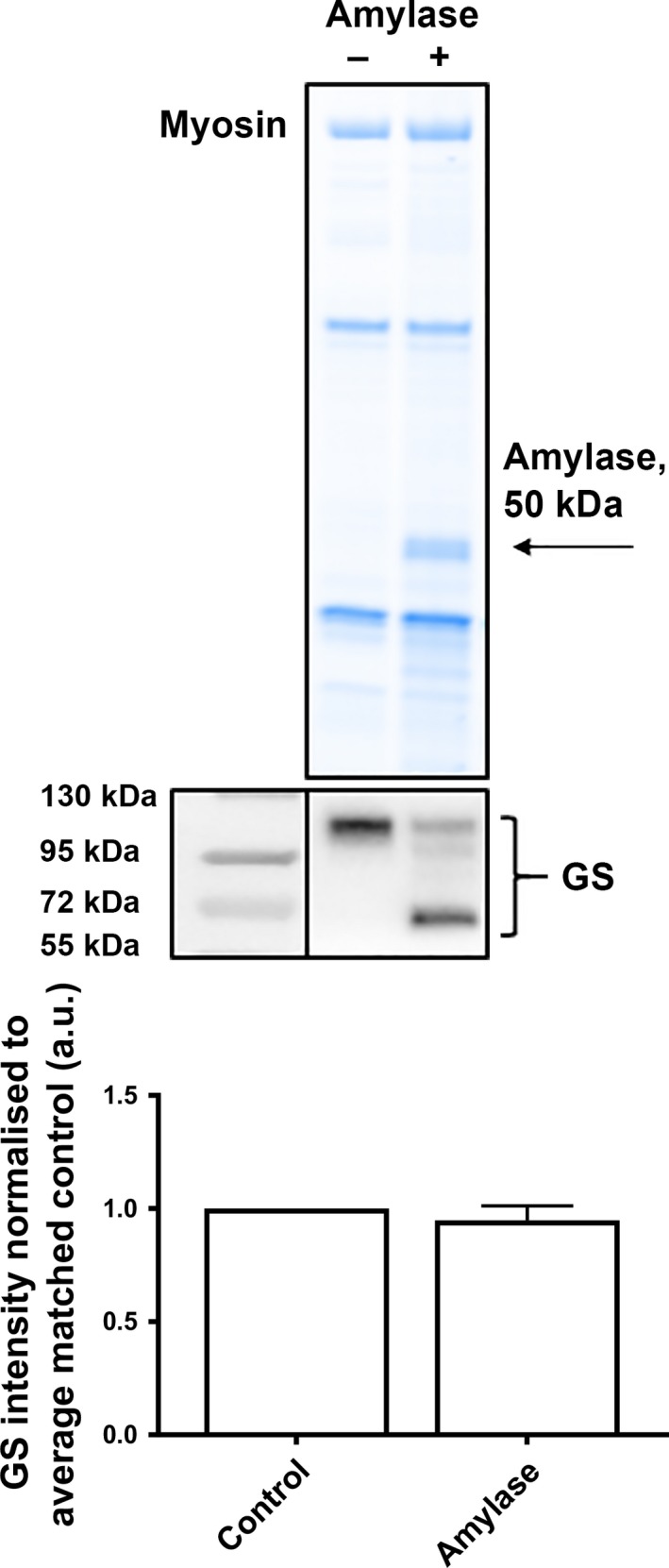
Effect of α‐amylase treatment on abundance of glycogen synthase in the EDL muscle detected by western blot. Myosin from the Stain Free gel indicative of total protein (*top),* representative western blot for glycogen synthase (GS, *middle*) and quantification of the abundance of GS from matching muscle homogenates prepared as normal (control) or with α‐amylase (amylase) treatment, expressed relative to control for a given sample pair, mean + SD, *n* = 5. Student's paired *t*‐test. GS, glycogen synthase

Treatment with α‐amylase resulted in the cleavage of GS protein in EDL muscle, and resulted in the appearance of several bands (Fig. 1). This cleavage was more evident in muscle from WT mice compared with *mdx* mice, and more evident in the 28 days compared with 70 days *mdx* mice (see later, Figs 4A, 6A). The collective intensity of bands seen in the amylase treated samples equated to the total GS protein abundance seen in the WT control. Figure 1 shows that there is no significant loss of GS protein with the α‐amylase treatment and that cleaved bands are indeed GS protein (Fig. 1). α‐Amylase had no effect on the appearance or abundance of any other glycogen handling protein investigated, as previously described Xu et al. (2015).

### Effect of taurine supplementation and age on skeletal muscle GLUT4, glycogenin protein content, and glycogen abundance

**Figure 2:**
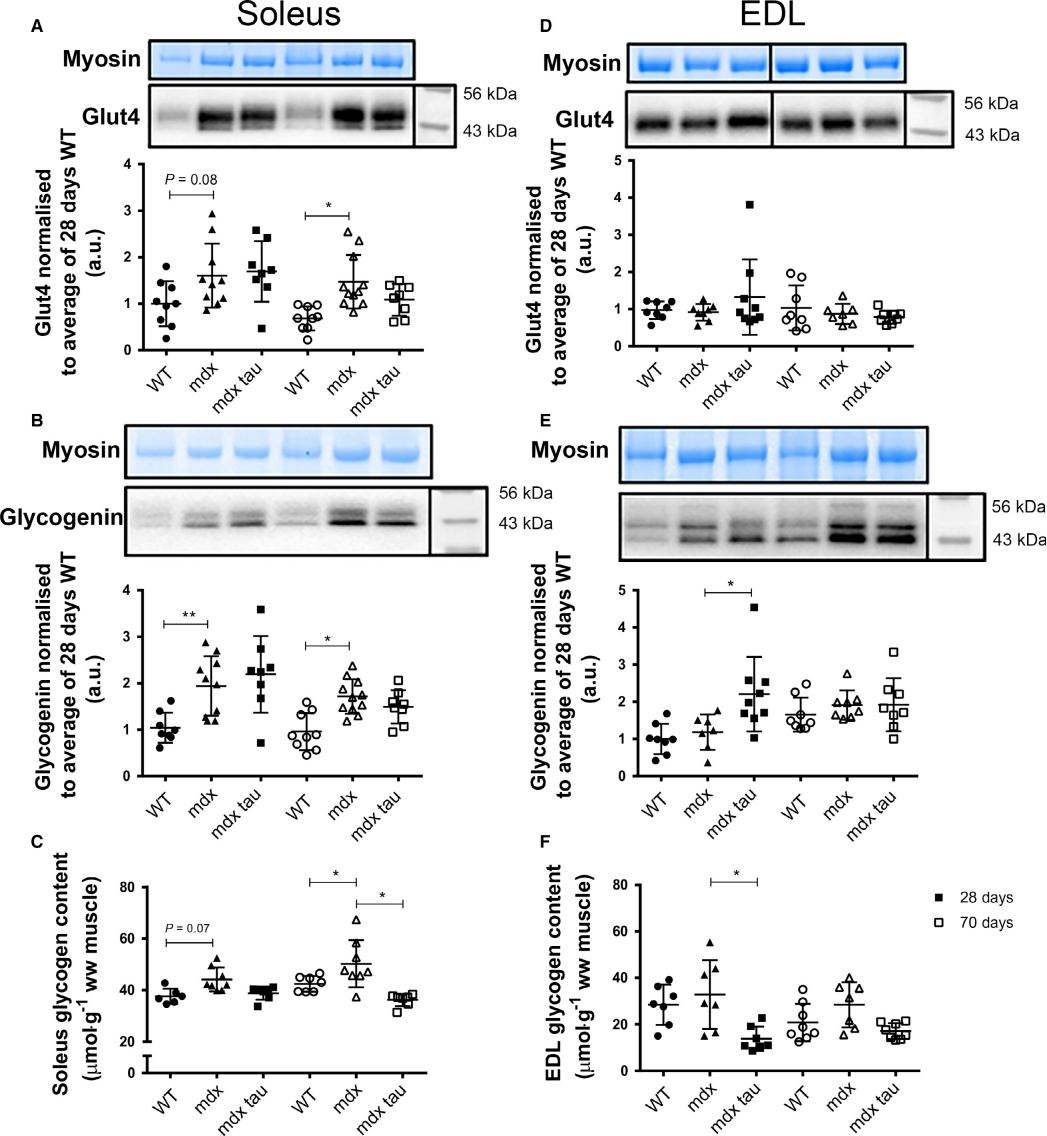
GLUT4 and glycogenin protein abundance, and glycogen content in the soleus and EDL muscles from 28 and 70 days WT, *mdx*, and *mdx* tau mice. (A) Myosin from the Stain Free gel, indicative of total protein (*top),* representative western blot for GLUT4 (*middle*), quantification of GLUT4 abundance (*bottom*) in the soleus muscles. (B) As for A, but for glycogenin. bottom band revealed after amylase treatment identified as glycogenin (data not shown). (C) Soleus glycogen content from 28 days (solid symbols) and 70 days (open symbols) WT (circles), *mdx* (triangles), and *mdx* tau (squares) mice. D, E, F as for A, B, C but EDL muscles. In (D) noncontiguous lanes from the same gel separated by black vertical lane. One‐way ANOVA with Holm–Sidak's post hoc analyses between relevant groups. Data presented as data points surrounding means ± SD, *n* indicated by number of symbols. Lines connecting different bars indicate significance at * *P *< 0.05, **P < 0.01 or *P*‐value shown.

Figure 2A shows there was a trend (*P *= 0.08) for an increase in GLUT4 protein abundance in the soleus muscle of 28 days *mdx* mice compared to the WT, and this persisted in 70 days *mdx* group with a 90% increase in GLUT4 protein abundance compared to the WT group. Figure 2B shows that soleus muscle glycogenin protein abundance was ~2‐fold greater in both 28‐ and 70‐day‐old *mdx* mice compared to the age‐matched WT control, with no difference observed between *mdx* and *mdx* tau mice at either age. Figure 2C shows there was a trend (*P *= 0.07) for an increase in glycogen content in the soleus muscle of 28‐ and 70‐day‐old *mdx* mice compared to the WT, and this persisted in the 70 days *mdx* groups compared to the WT group. A reduction in soleus muscle glycogen content was observed in *mdx* tau mice compared to *mdx* mice (Fig 2C). No group or age‐related differences in the abundance of GLUT4 was observed in the EDL (Fig 2D). There was an approximate 70% increase in the amount of glycogenin protein abundance in the EDL muscle from 28 days *mdx* tau mice compared to the *mdx* (Fig 2E), although 28 days *mdx* tau mice showed a 50% decrease in muscle glycogen content compared to the *mdx* despite this increase (Fig. 2E). There was no difference in abundance of glycogenin protein or glycogen content in EDL muscles between 70 days groups (Fig. 2E and F).

### PAS stain suggests similar skeletal muscle glycogen content in 28 and 70 days WT, *mdx* and *mdx* tau EDL and soleus muscles

**Figure 3:**
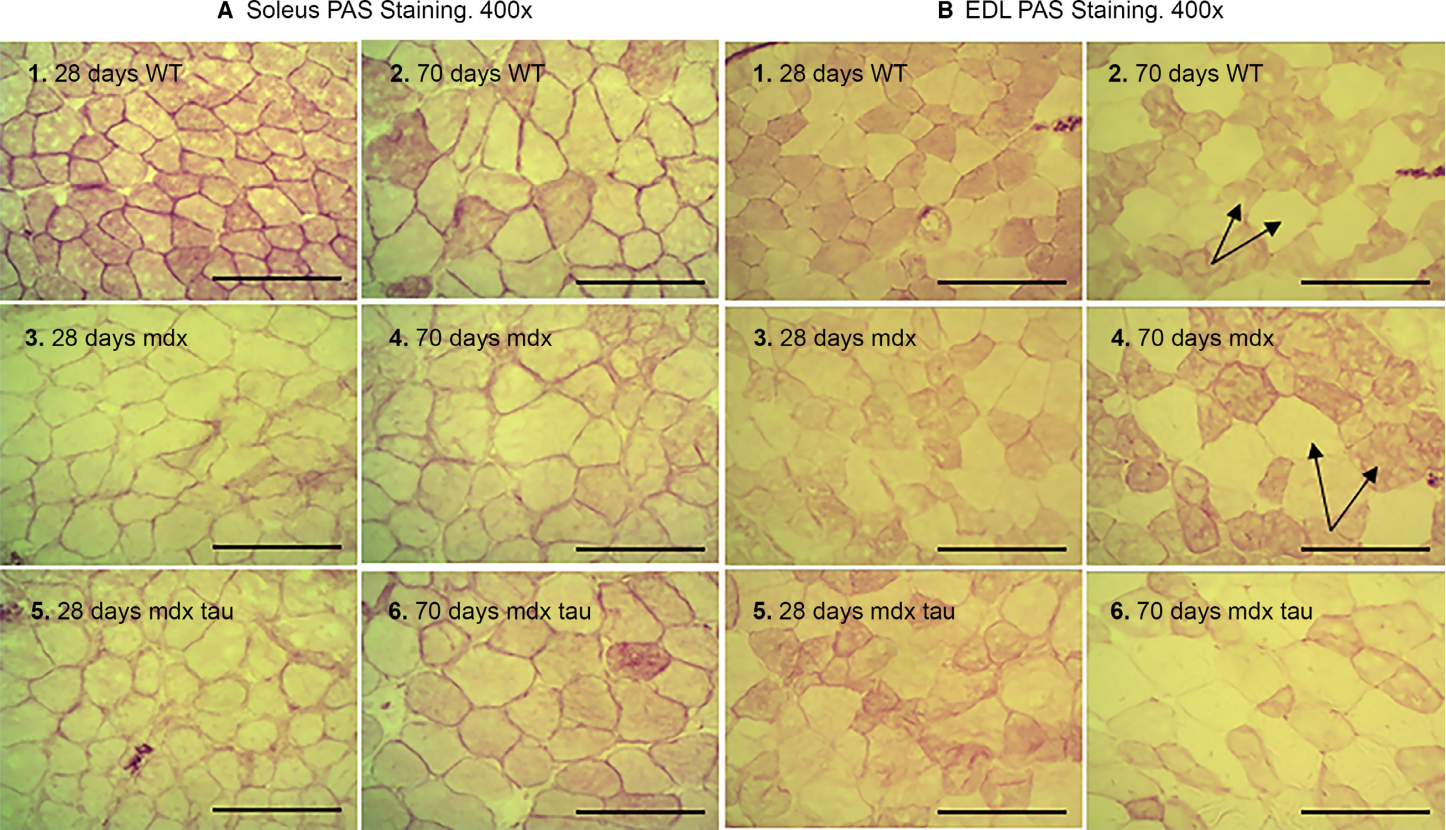
Relative skeletal muscle glycogen content as visualized by PAS stain. PAS stain of 10 *μ*m cross‐sections of 28 and 70 days WT, *mdx*, and *mdx* tau soleus (sol, left) and EDL (right) muscles shown at 400× magnification. All images shown from each muscle (EDL or soleus) were stained simultaneously (see Methods). Darker colored muscle fibers indicate greater glycogen content – see arrows indicating a darkly stained fiber next to a weakly stained fiber. Scale bar = 100 *μ*m, *n *= 5. PAS, Periodic acid–Schiff

PAS staining can be used to provide a visual and qualitative assessment of glycogen content, where the more intensely pink‐stained fibers and/or muscles contain more glycogen (Fig. 3). It can be seen that the staining is often heterogeneous both within and between groups and ages, with some fibers containing more glycogen than others (e.g. see arrows in panels B2 and B4 in Fig. 3).

### Elevated p‐GS with age in the soleus muscles of *mdx* mice is not attenuated by taurine supplementation

**Figure 4:**
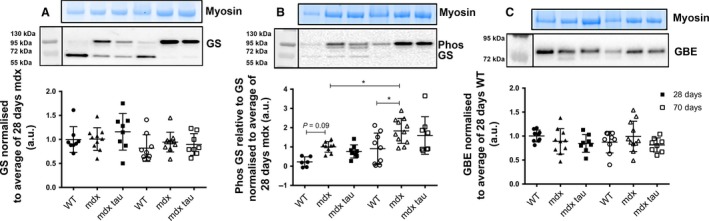
Anabolic glycogen associated protein abundance in the soleus muscle of 28 and 70 days WT, *mdx*, and *mdx* tau mice. Shown for each panel is the myosin from the Stain Free gel, indicative of total protein (*top),* the representative western blot protein (*middle*), and quantification of protein abundance (*bottom*) from 28 days (solid symbols) and 70 days (open symbols) WT (circles), *mdx* (triangles), and *mdx* tau (squares) mice. (A) Glycogen synthase (GS), (B) phosphorylated GS (p‐GS), (C) glycogen branching enzyme, each expressed relative to average of the 28 days WT run on a given gel. One–way ANOVA with Holm–Sidak's post hoc analyses between relevant groups. Data presented as data points surrounding means ± SD, *n* indicated by number of symbols. Lines connecting different bars indicate significance at *P *< 0.05 or *P*‐value as shown.

There were no age‐specific or group differences in the abundance of soleus muscle GS protein (Fig. 4A). Similarly, despite a trend (*P* = 0.09) for increased p‐GS in 28 days *mdx* compared to WT mice, there was no difference in the abundance of relative p‐GS between 28 days groups (Fig. 4B). *mdx* mice (70 days ) had a twofold increase in the relative abundance of p‐GS compared to either the age‐matched WT and the 28 days *mdx* (Fig. 4B), indicating less GS in the active state in these 70 days animals. There were no age or group differences in the abundance of GBE protein (Fig. 4C).

### Effect of taurine supplementation and age on soleus muscle catabolic glycogen‐related protein abundance

**Figure 5:**
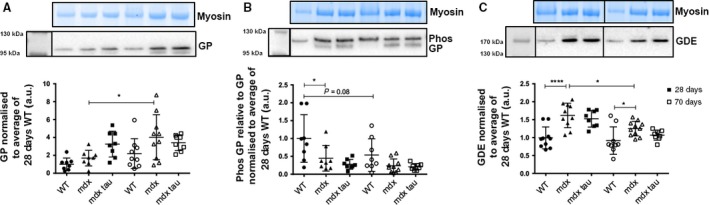
Catabolic glycogen‐associated protein abundance in the soleus muscle of 28 and 70 days WT, *mdx*, and *mdx* tau mice. Shown for each panel is the myosin from the Stain Free gel, indicative of total protein (*top),* the representative western blot protein (*middle*), and quantification of protein abundance (*bottom*) from 28 days (solid symbols) and 70 days (open symbols) WT (circles), *mdx* (triangles), and *mdx* tau (squares) mice. Protein abundance expressed relative to the average of the 28 days WT run on a given gel. (A) Glycogen phosphorylase (GP), (B) phosphorylated glycogen phosphorylase (p‐GP), and (C) glycogen debranching enzyme (GDE). In (C), noncontiguous lanes from the same gel separated by black vertical lane. Note the GP and p‐GP probes were after GS which is still seen as the lower band in A and B. One‐way ANOVA with Holm–Sidak's post hoc analyses between relevant groups. Data presented as data points surrounding means ± SD, *n* indicated by number of symbols. Lines connecting different bars indicate significance at *P < 0.05, ***P < 0.001 or *P*‐value as shown.

No difference was observed in soleus muscle GP protein abundance between 28 and 70 days WT groups; however, the 70 days *mdx* group had a ~2‐fold increase in GP protein abundance compared to the 28 days *mdx* group (Fig. 5A). There was a 55% decrease in relative p‐GP protein abundance between the 28 days WT and age‐matched *mdx* mouse, and a trend (*P* = 0.08) for reduced p‐GP abundance with increasing age in WT mice (Fig. 5B). EDL p‐GP protein abundance was not influenced by taurine (Fig. 5B). No further group or age‐specific differences in p‐GP abundance were observed. Both 28‐ and 70‐day‐old *mdx* mice had ~60% and 40% more GDE protein abundance, respectively, than their age‐matched WT groups (Fig. 5C), and 70 days *mdx* mice had approximately 20% less GDE than the 28 days *mdx* group (Fig. 5C).

### No group or age‐related difference in the abundance of glycogen‐associated proteins in the EDL muscle of 28 and 70 days WT, *mdx*, and *mdx* tau mice

**Figure 6:**
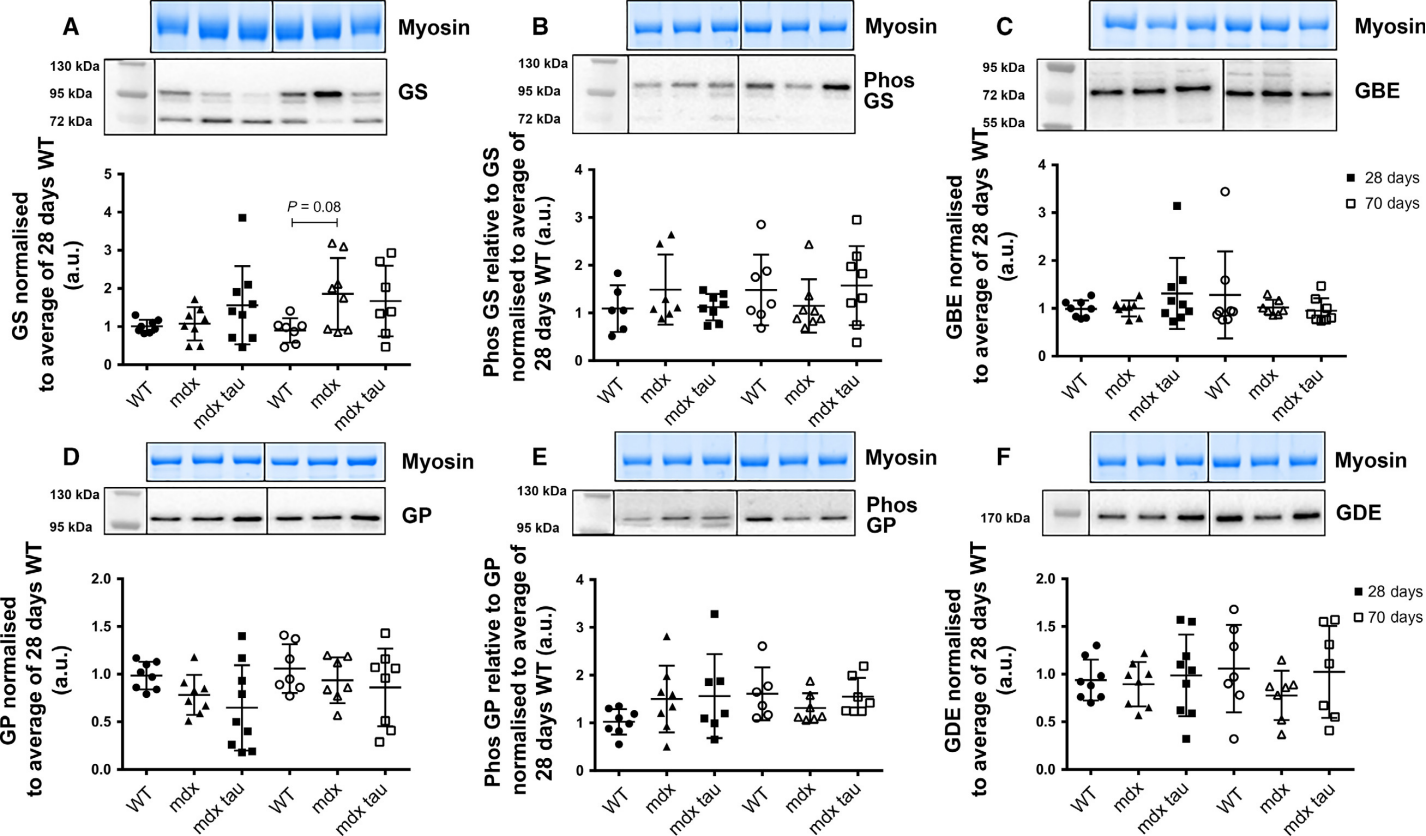
Abundances of glycogen‐associated proteins in the EDL muscle from 28 and 70 days WT, *mdx*, and *mdx* tau mice. Shown for each panel is the myosin from the Stain Free gel, indicative of total protein (*top),* the representative western blot protein (*middle*), and quantification of protein abundances (*bottom*) from 28 days (solid symbols) and 70 days (open symbols) WT (circles), *mdx* (triangles), and *mdx* tau (squares) mice. Phosphorylated proteins are expressed relative to its total protein, that is glycogen synthase (GS) or glycogen phosphorylase (GP) (A) GS, B. phosphorylated GS (p‐GS), (C) glycogen branching enzyme (GBE), (D) GP, (E) phosphorylated GP (p‐GP). and (F) glycogen debranching enzyme (GDE). Noncontiguous lanes from the same gel separated by black vertical lane. Protein abundance expressed relative to the average of 28 days WT. One‐way ANOVA with Holm–Sidak's post hoc analyses between relevant groups. Data presented as data points surrounding means ± SD, *n* indicated by number of symbols. Significance at *P *< 0.05.

In the EDL muscle, there were no group or age‐related differences observed in the abundance of GS, p‐GS, GBE, GP, p‐GP, and GDE proteins in both 28 and 70 days WT, *mdx* and *mdx* tau mice (Fig. 6A–E). Data shown to visualize the variability in protein abundance within respective groups.

## Discussion

In this study, we performed experiments to investigate the reports of aberrant glycogen metabolism in the *mdx* mouse model of DMD. We comprehensively investigated the abundance of glycogen and its associated proteins taking into consideration the implications of disease stage, muscle type, and taurine supplementation. We found an increase in *mdx* mouse skeletal muscle glycogenin protein abundance and glycogen content, and differences in the abundance of glycogen‐associated proteins p‐GS, p‐GP and GDE that were predominantly specific to the more oxidative soleus muscle. We further identified that age and therefore disease severity did not influence glycogen protein abundance and that taurine supplementation affords no appreciable benefit to glycogen metabolism.

### Analysis of glycogen‐associated proteins in the 28‐ and 70‐day‐old *mdx* mice

As the primary mediator of insulin and contraction‐induced glucose transport into the skeletal muscle, GLUT4 plays a vital role in whole body glucose homeostasis, translocating to the cell plasma membranes and facilitating glucose uptake into the cell (Hansen et al. 1995; Thorens and Mueckler 2010). In the soleus muscle of both 28‐ and 70‐day‐old *mdx* mice, GLUT4 protein abundance was elevated when compared to the WT (Fig. 2A), indicating an increased ability for glucose uptake in these animals. This coincided with a twofold greater abundance of glycogenin protein in the soleus muscle of 28‐ and 70‐day‐old *mdx* mice compared to WT controls (Fig. 2B). The increases in both GLUT4 and glycogenin protein theoretically increase the rate of glucose entry and the potential to synthesize skeletal muscle glycogen. However, this did not seemingly translate into a proportional increase in glycogen content, with only a modest increase in soleus muscle glycogen content observed in both 28‐ and 70‐day‐old *mdx* mice (Fig. 2C). Glucose intolerance has previously been reported in *mdx* mice (Stapleton et al. 2014), and the increased abundance of GLUT4 protein despite modestly elevated glycogen content may suggest aberrant GLUT4 function in the *mdx* mouse. Interestingly, however, this was specific to the more oxidative soleus muscle, with no alterations observed in more glycolytic EDL muscle. An alternate hypothesis is that while *mdx* mice have an increased abundance of glycogenin; this could translate into a greater number of glycogen granules, which are smaller but contain the same amount of glucose moieties overall. Finally, it is possible that there was an insufficient dietary carbohydrate to facilitate the storage of excess glucose as glycogen, but that *mdx* and WT mice had comparable glycogen levels despite reports of greatly increased *mdx* energy requirements makes this unlikely (Radley‐Crabb et al. 2014).

Previously *mdx* mice have shown decreased levels of liver glycogen and an approximate 50% increase in skeletal muscle glycogen at 16 weeks of age (Stapleton et al. 2014). While not observed to that extent here, the significant increase in GLUT4 and glycogenin expression observed may also be an adaptation to ensure greater glucose partitioning to the skeletal muscle for use locally. This may aid in regeneration events, as opposed to storage in the liver where glucose maintains the homeostasis of the organism as a whole. Previously, Radley‐Crabb et al. (2014) identified that both juvenile (4–5 weeks) and adult (12–14 weeks) *mdx* mice had increased energy demands compared to WT controls, specifically with regard to protein turnover and muscle regeneration that may be responsible for altered partitioning of glucose storage seen here.

Slow‐twitch oxidative muscle fibers, more predominant in soleus muscles, are less preferentially affected by contraction‐induced damage in dystrophy, and the upregulation of GLUT4 and glycogenin may be a metabolic adaptation to ensure glucose availability allowing energy for regeneration, thereby assisting muscle longevity in this model (Webster et al. 1988; Selsby et al. 2012). In support of this, Carnwath and Shotton (1987) identified a progressive increase in the proportion of type 1 muscle fibers from the soleus muscle of *mdx* mice with age, and that the proportion of these fibers that persisted through to adulthood was significantly greater than type 2 fibers. Olichon‐Berthe et al. (1993) identified an increase in GLUT4 protein abundance in unspecified hind limb skeletal muscle from *mdx* mice at both 5–6 weeks and 6–7 months of age. In that study, however, muscle samples were centrifuged which can be a problem when attempting to quantify specific proteins (Mollica et al. 2009; Murphy and Lamb 2013). More recently, Stapleton et al. (2014) found a twofold increase in *mdx* glycogenin expression, and while sample preparation details were not provided, normalization of the protein was made to glyceraldehyde‐3‐phosphate dehydrogenase (GAPDH) (Stapleton et al. 2014), which is an inappropriate loading control for the quantification of protein abundance in skeletal muscle due to its variability with both age and fiber type, at least in humans (Wyckelsma et al. 2016). As such, any loading control outside of total protein needs to be validated. It is further difficult to directly compare that study with the current work because details of the specific muscles examined were not provided and it is evident that glycogen content and proteins are muscle type dependent (Murphy et al. 2012; Xu et al. 2015).

Interestingly using qualitative biochemical techniques, Stapleton et al. (2014) identified a significant increase in the glycogen content of mixed muscle quadriceps and fast‐twitch tibialis anterior (TA) muscle from 16‐week‐old *mdx* mice, and an increase in both slow‐ and fast‐twitch glycogen content (Shown as type 2A and 2B/X) determined by optical density of PAS stain. PAS staining can only be used as a qualitative indication of fiber‐type glycogen distribution because the intensity of color cannot be calibrated (Schaart et al. 2004). Even so, the intensity of color indicates more glycogen content and can therefore distinguish between glycolytic as opposed to oxidative muscle fibers.

While glycogen granules are formed by the autoglucosylation of glycogenin by the synergetic actions of GS and GBE proteins, we found no increase in the abundance of these anabolic enzymes in the soleus and EDL muscles of either 28 or 70 days *mdx* mice compared to WT controls (Figs 4A,C and 5A and C). In 16‐week‐old *mdx* mice, Stapleton et al. (2014) reported an ~70% decrease in the activity of GBE (expressed as AU/*μ*g protein) in the unspecified skeletal muscle compared to the WT, suggesting that the increase in skeletal muscle glycogen was compensatory for the poorly branched glycogen molecules this would produce. We offer an alternate hypothesis that an endogenous abundance of GS and GBE proteins, despite increased GLUT4 and glycogenin, may suggest these enzymes are not rate limiting at both 28 and 70 days of age. GS is activated by the allosteric stimulator gluccose‐6‐phosphate (G6P) and dephosphorylation via the actions of kinase‐3 and insulin (Bouskila et al. 2010). Here, we measured the relative abundance of the inactive form of GS, p‐GS, and found no difference across 28 days groups (Figs 4B,6B), suggesting normal activity of GS giving no cause to assume *mdx* mice have issues with glycogen storage. That 70 days *mdx* mice have a twofold increase in relative p‐GS than both the age‐matched WT control and the 28 days *mdx* mice in the soleus muscle, indicates that there is less GS in the active state contributing to glycogen building in the soleus muscle, although the cause for this is unknown.

During energy requiring events where circulating glucose is insufficient, the skeletal muscle mobilizes the catabolic enzymes GP and GDE to liberate glucose from intramuscular glycogen stores for use in ATP synthesis. While there was no difference in the abundance of soleus muscle GP protein between *mdx* and WT mice at either 28 or 70 days of age, GP protein abundance was significantly increased with age in *mdx* mice (Fig. 5A). However, when looking at the active state of GP and p‐GP, the 28 days WT showed a greater ability to break down α‐1,4‐glycosidic linkages than the age‐matched *mdx* group that did not persist with age. Similar to the 16‐week‐old *mdx* mice investigated by Stapleton et al. (2014), we found increased abundance of GDE in *mdx* mice at both 28 and 70 days, although 70 days *mdx* mice had reduced GDE in the soleus muscle when compared to younger 28 days *mdx* mice (Fig. 5C). The findings that the abundance and theoretically the activity of GP, the enzyme responsible for the degradation of glycogen branch points, is relatively greater than the more abundant and exposed glucose residues attached by a‐1,4‐glycosidic linkages is curious. This may point toward an altered glycogen structure in *mdx* mice, although this remains highly speculative. Interestingly, the EDL exhibited no change in the abundance of any catabolic glycogen associated proteins.

### Impact of taurine on glycogen metabolism in the 28‐ and 70‐day‐old *mdx* mice

Previously, we identified that supplementation with the amino acid taurine increased in situ tibialis anterior strength and improved visible muscle health in 28 days *mdx* mice (Barker et al. 2017). To elucidate whether the beneficial effects of taurine could in part be attributed to enhanced glycogen metabolism, we investigated the aforementioned compliment of proteins. While taurine supplementation elevated glycogenin protein expression in the EDL muscle of 28 days *mdx* mice, curiously glycogen content was significantly reduced compared to the non‐taurine supplemented *mdx* (Fig 2E and F). This is in contrast to previous findings in the similarly fast‐twitch *tibialis anterior* muscle of nondystrophic WT mice where taurine supplementation facilitated glycogen repletion postexercise (Takahashi et al. 2014, 2016). Glycogen content was also reduced in the soleus muscle of the 70 days *mdx* tau mouse. Why taurine would result in a decrease in glycogen content despite an increase in glycogenin and no appreciable difference in the phosphorylation status of GS or GP is unknown.

A possible explanation is the degradation of glycogen by an alternate pathway known as glycophagy (Roach et al. 2012). Stapleton et al. (2014) identified a twofold increase in the abundance of starch‐binding domain protein 1 (STBD1), a protein involved in the glycophagy pathway proposed to degrade incorrectly branched glycogen, in *mdx* compared to WT mice. Curiously, in that same study, the increase in STBD1 was associated with a similar increase in skeletal muscle glycogenin and glycogen content. Why increased STBD1, which is proposed to remove glycogen, would coincide with an increase in glycogen content is unclear. In the current, we found no difference in EDL glycogenin and glycogen content between *mdx* and WT mice, potentially indicating taurine is facilitating an alternate pathway of glycogen breakdown. If this aids the pathology of the *mdx* mouse by freeing glucose for use in ATP‐consuming pathways remains unclear.

## Conclusion

The *mdx* mouse exhibits a remarkable ability to regenerate skeletal muscle into adulthood that ultimately rescues the animal from the severe degeneration seen at 3–4 weeks of age. The finding from this study is that a slow‐twitch muscle‐specific alteration in proteins associated with glucose storage may be an adaptation to meet the constant energy demand associated with cycles of muscle degeneration/regeneration, but the recovery of the *mdx* mouse with age does not appear to be facilitated by an alteration in glycogen handling. We further identify that the benefit seen with taurine supplementation in the 28 days *mdx* mouse model of DMD described previously (Barker et al. 2017), similarly does not appear to be derived from an improvement in glycogen handling.

## Conflict of Interest

We hereby disclose that there are no conflict of interests or governing funding bodies associated with this study.
